# Paradigms of convergent evolution in enzymes

**DOI:** 10.1111/febs.17332

**Published:** 2024-11-22

**Authors:** Ioannis G. Riziotis, Jenny C. Kafas, Gabriel Ong, Neera Borkakoti, António J. M. Ribeiro, Janet M. Thornton

**Affiliations:** ^1^ European Bioinformatics Institute (EMBL‐EBI) Cambridge UK

**Keywords:** active site, convergence, enzyme, evolution, mechanism

## Abstract

There are many occurrences of enzymes catalysing the same reaction but having significantly different structures. Leveraging the comprehensive information on enzymes stored in the Mechanism and Catalytic Site Atlas (M‐CSA), we present a collection of 34 cases for which there is sufficient evidence of functional convergence without an evolutionary link. For each case, we compare enzymes which have identical Enzyme Commission numbers (i.e. catalyse the same reaction), but different identifiers in the CATH data resource (i.e. different folds). We focus on similarities between their sequences, structures, active site geometries, cofactors and catalytic mechanisms. These features are then assessed to evaluate whether all the evidence for these structurally diverse proteins supports their independent evolution to catalyse the same chemical reaction. Our approach combines published literature information with knowledge‐based computational resources from, amongst others, M‐CSA, PDBe and PDBsum, supported by tailor‐made software to explore active site structures and assess similarities in mechanism. We find that there are multiple types of convergent functional evolution observed to date, and it is necessary to investigate sequence, structure, active site geometry and enzyme mechanisms to describe such convergence accurately.

## Graphical abstract

Some enzymes are able to catalyse the same reaction despite possessing different structures. Here, we used a combined strategy of analysing published literature and knowledge‐based computational resources to explore this. We conclude that multiple varieties of convergent functional evolution exist to date, but that it is necessary to investigate sequence, structural features, active site geometry and enzyme mechanisms to accurately identify cases with such convergence.

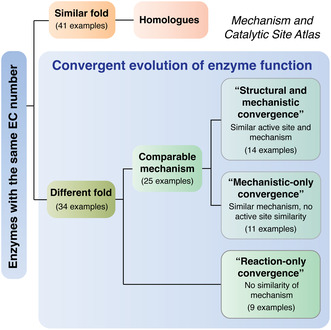

**Abbreviations**
3D: three‐dimensional
CATH: class, architecture, topology, homology
CoA: coenzyme A
EC: Enzyme Commission
GH: glycosyl hydrolase
M‐CSA: Mechanism and Catalytic Site Atlas
NAD: nicotinamide adenine dinucleotide
PDB: Protein Data Bank
PDBe: Protein Data Bank in Europe
PLP: pyridoxal phosphate
RMSD: root mean square deviation

## Introduction

Nature shapes biological macromolecules during evolution, allowing mutable elements to change, or conserving them by natural selection. The extent of selective pressure is variable, and this contributes to functional divergence in proteins of common ancestry. We have recently reviewed some of the concepts in enzyme evolution, especially functional divergence from a mechanistic viewpoint [1]. All known enzyme reactions are performed by a relatively limited number of structural folds. However, the chemical reaction space is vast and there may be many biological reactions we have yet to discover. Despite this, amongst the many reactions we know, many are catalysed by more than one family of enzymes that are not related by evolution. Surprisingly, this is quite common, with a reaction on average being catalysed by ~ 2 evolutionarily unrelated enzymes [2, 3]. Such analogues are called isofunctional enzymes or isozymes [4] and the evolutionary phenomenon that describes them is convergent evolution [5].

It is not uncommon for a catalytic process, or part of it, to be facilitated by a set of chemical groups in a well‐defined geometry [6]. These 3D constellations often drive a unique sequence of events of the catalytic mechanism. Popular examples are the various catalytic triads in proteinases [7, 8] that hydrolyse peptide bonds by nucleophilic substitution. Although the nucleophile position might be occupied by different residues, usually Ser or Cys, the spatial arrangement of the triad (Nucleophile‐His‐Acid) is highly similar amongst most proteinases and has evolved independently in analogues [9]. In all cases, the same sequence of catalytic steps takes place. Similarly, the binding affinity for a substrate depends on the geometry of residues in the binding pocket, where any conformational changes can dramatically affect ligand selectivity. In the case of small ligands (especially metal ions), binding constraints can be strong, leading to a common geometry in unrelated proteins (e.g. Fe‐SO metal cluster binding site).

In the context of convergent evolution, we can ask questions such as:

1. Since functional convergence can occur in multiple ways, what are the different facets of such convergence?
2. Can we distinguish different paradigms, and which occur most often in nature?
3. Why has nature evolved the same function more than once?

Leveraging the integrated information within the Mechanism and Catalytic Site Atlas (M‐CSA)—that is, sequence, structure, homologues, functional annotation of residues, curated annotation of catalytic residues and explicit description of the mechanism—herein we aim to address these questions and define broad paradigms of enzyme convergent evolution.

We present 34 detailed examples of soluble enzyme analogues (pairs or triplets), characterising their catalytic sites and mechanisms to illustrate convergent evolution of function. We also provide evidence that categorisation is not straightforward, and in several cases, enzymes adhere to multiple paradigms. Our observations highlight some limitations in enzyme function and structure classification systems, and the need to consider additional parameters such as local active site geometry, cofactor selectivity and chemistry to infer ambiguous evolutionary relationships. This systematic analysis of multiple enzyme features (sequence, local and global structure, catalysed reaction, catalytic mechanism, substrate selectivity, promiscuity, etc.), provides extra knowledge, useful for enzyme design by computational methods and directed evolution [10].

## Results

**Fig. 1:**
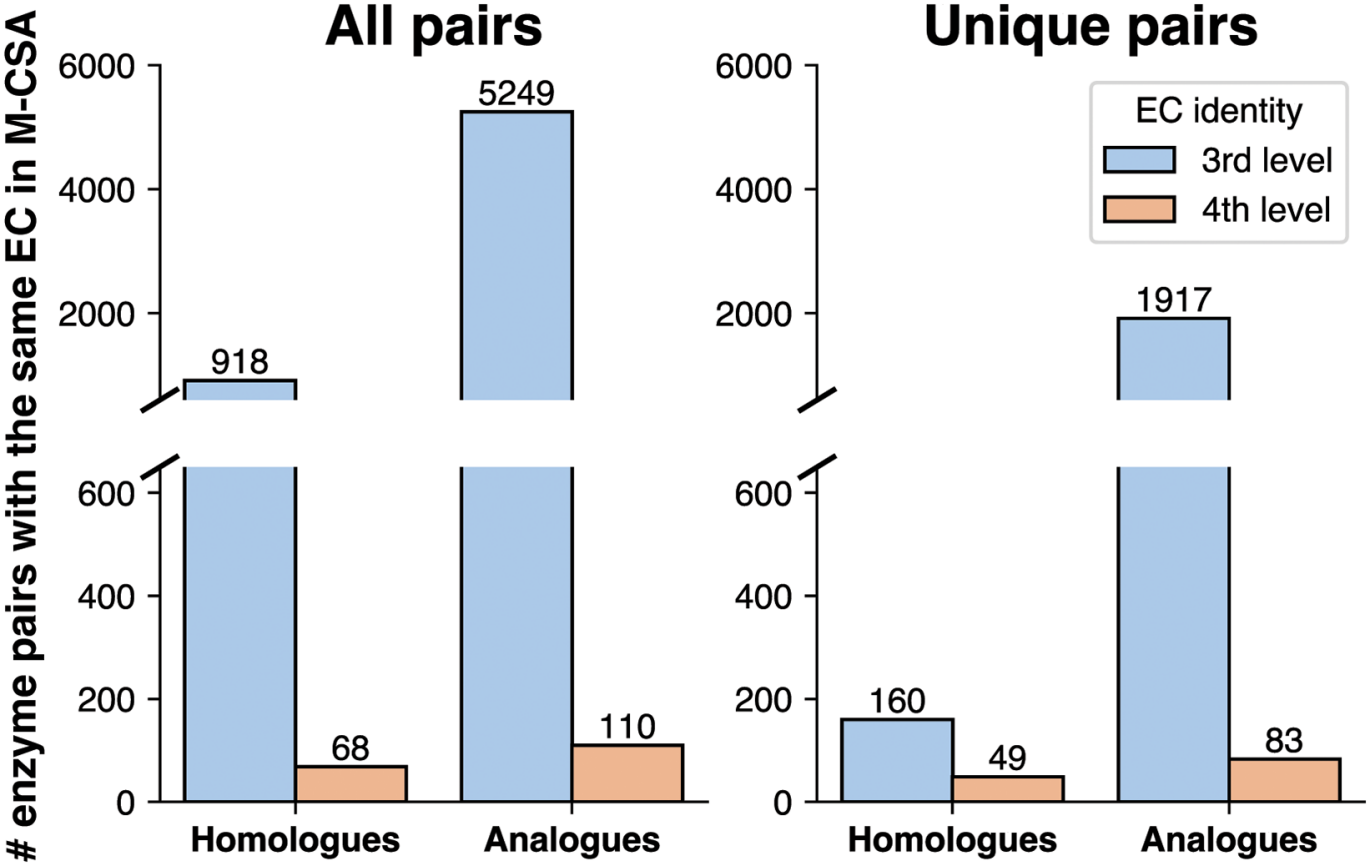
Number of enzyme pairs in M‐CSA sharing the same EC number, when looking at the sub‐subclass (3rd number) or sub‐sub‐subclass (4th number) level. Homologues have identical catalytic domain CATH numbers and analogues have different CATH numbers. Since M‐CSA is redundant in EC and CATH (multiple entries with the same numbers), we further filtered for ‘unique’ pairs, with their numbers shown on the right panel.

**Fig. 2:**
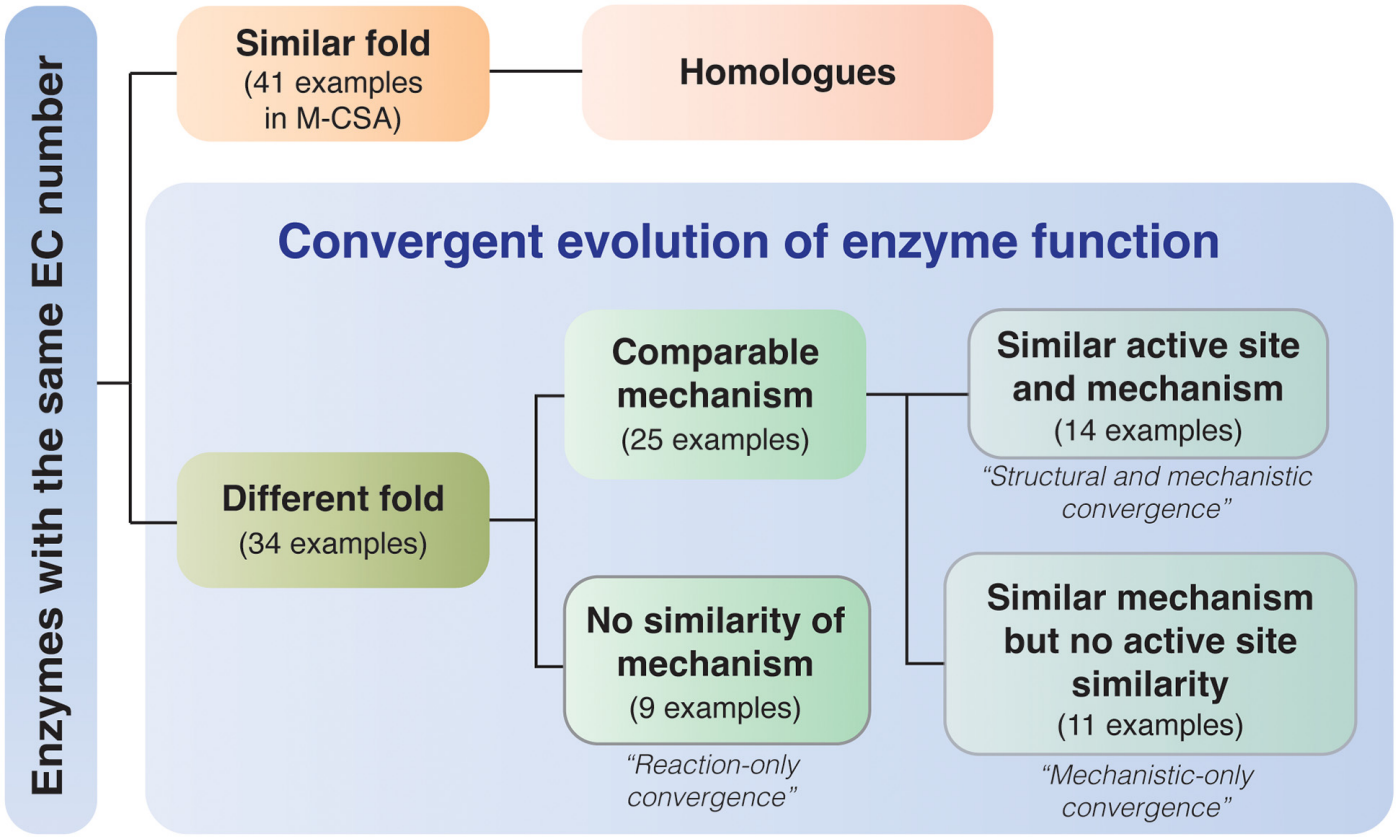
Evolutionary relationships of enzymes of the same function. This is a summary of enzyme pairs that catalyse the same reaction, deposited in the Mechanism and Catalytic Site Atlas. These pairs are split into homologous or nonhomologous (convergent), and the latter are further grouped according to the level of similarity (active site structure and/or mechanism). Paradigms of convergence are quoted.

The Mechanism and Catalytic Site Atlas (M‐CSA) aims to include entries for all known enzyme reactions for which sequence, structure and functional data are available, and so inevitably the content reflects the protein coverage in UniProt and PDB, which are both biased towards well‐studied enzymes. The rationale of M‐CSA curation is broadly based on including one or more entries for every unique EC number, where each entry is an enzyme catalysing the reaction of that EC number with a different mechanism. We took advantage of this redundancy to identify and compare pairs of enzymes, which appear to perform the same reaction but belong to different structural families (i.e. examples of convergent evolution). To this end, we extract two datasets of paired enzymes: (a) all pairs of entries in M‐CSA (2021 update) with the same EC number at reaction level (sub‐sub class—EC x.x.x.‐), and (b) all pairs of entries with the same EC number performing the same reaction on the same substrate (sub‐sub‐subclass—EC x.x.x.x). In a pair, the two enzymes might be homologues (their catalytic domains belonging to the same CATH superfamily) or analogues (when the proteins are from different CATH superfamilies, thus are likely to have evolved independently). The complete dataset consisted of 6345 pairs, many of which occur more than once. For example, M‐CSA contains six entries for β‐lactamases (EC 3.5.2.6), three of which belong to CATH superfamily 3.40.710.10 and the other three to 3.60.15.10. Most of the duplicates differ in their mechanism, and a few are attributed to curation mistakes. We reduced this redundancy by further filtering for unique analogue pairs (as per EC and CATH), lowering the number of pairs to 2209. Using CATH numbers as filters we distinguished homologous pairs (identical CATH number) from analogous pairs (different CATH numbers). Figure 1 shows the comparison of the two datasets. We find many more analogues than homologues (only 9–15% are homologues, covering 41 EC numbers in total) reflecting the content of the M‐CSA. We also find that considering only the reaction at the third level (i.e. ignoring substrate specificity by removing the 4th EC level filter) leads to a steep increase in the number of enzyme pairs. This increase remains significant even in unique pairs (right panel of Fig. 1). However, we already know that in nature, most new functions evolve from old functions [11] by simply changing the substrate—but here we are deliberately targeting pairs of enzymes with the same function but in different structural families—that is, analogues. Out of these sets, we selected the unique analogous pairs matching at the 4th EC level (83 pairs in total, with both members of the pair catalysing the same reaction with identical substrates). After filtering (see Materials and methods) we ended up with a curated set of 34 pairs/triplets that exemplify three paradigms of convergence (Fig. 2).

These 34 examples were examined for similarities in different dimensions, some of which we discussed in the context of bioinformatics in our recent review [1]. Using computational tools and/or literature reviewing, we looked at the following:

1. overall structural similarity (CATH superfamily assignment and 3D fitting),
2. active site structural similarity (quantified by RMSD of aligned residues),
3. mechanistic features (catalytic steps [12, 13] or/and residue roles),
4. substrate similarity,
5. cofactor presence/similarity.

Our results are organised as pairs or triplets of analogues in Tables 1, 2, 3. Three selection rules are implicit in this survey of convergent evolution: (a) catalytic domains should always belong to different CATH superfamilies and (b) all four EC numbers should be identical and (c) analogues should share at least one identical product or substrate. This means that different paradigms of evolutionary convergence are defined by similarities in the active site composition and geometry, catalytic mechanism and cofactor selectivity. Focussing solely on mechanism, three general ‘cases’ are observed. First, analogues that are completely different in their mechanism of catalysis. In the second ‘case’, analogues that are similar but only part of the reaction is the same (e.g. phospholipases (EC 3.1.1.4) had similarities in steps 1 and 2 of one enzyme and steps 3 and 4 of the other respectively). In the third case, mechanisms between the analogues are essentially the same, with only minor differences. For these, the mechanisms are nearly identical except for differences in the catalytic residues being used (e.g. both analogous Papain‐like cysteine proteases (EC 3.4.22.28) use a Cys‐containing catalytic triad, but the His activator can be Asp or Asn). Combining these mechanistic paradigms with any localised structural similarities in the active site [14], led us to define three general paradigms of convergence in enzymes, described in the following section.

**Table 1:** Analogous enzyme examples with structural and mechanistic convergence.

Enzyme	EC	CATH domain	M‐CSA ID	PDB ID	Residue composition	3D‐aligned residues	RMSD (Å)	Mech. sim. (calc. perc.)	Cofactor	Notes	Refs.
Carbonate dehydratase	4.2.1.1 (a)	2.160.10.10	516	1QRM	EEHHHNQ	His117 Glu62 His122 His81	1.45	Yes (30%)	Co^3+^	Similar metal binding site. Sulphate moieties bind close to HCO_3_ positions in both analogues.	[15, 16]
		3.10.200.10	216	5GMN	EHHHHT	His94 Glu106 His119 His96			Zn^2+^		
Εndonuclease	3.1.21.1	3.90.540.10	791	2GZI	EHHHHRR	His103 His127 Glu100	1.43	Yes (30%)	Zn^2+^	Similar initial steps of nucleophilic attack on phosphate via a proton relay. A Glu‐His dyad is present in both analogues.	[17]
		3.60.10.10	41	2A40	DDEEHHY	His252 His134 Glu78			Mg^2+^		
Ornithine decarboxylase	4.1.1.17	3.40.640.10	860	1ORD	DHK	Lys355 His223	1.75	Yes (n/a)	PLP	Identical mechanism between bacterial/mammalian analogues. Their only difference is the mirrored arrangement of residues.	[18, 19]
		3.20.20.10	937	5GJO	EHK	Lys51 His179			PLP		
Cytosine deaminase	3.5.4.1	3.20.20.140	710	1K6W	DEHHHQ	His214 Glu217	0.69	Yes (35%)	Zn^2+^	In both analogues, His ligates a metal. Glu deprotonates a water molecule and protonates cytosine N3. Difference lies in the combination of metal‐ligating residues.	[20, 21]
		3.40.140.10	636	1UAQ	CCEHS	His62 Glu64			Zn^2+^		
Protein tyrosine phosphatase	3.1.3.48	3.40.50.2300	462	2P4U	CCDNRS	Ser20 Cys13 Arg19	1.30	Yes (n/a)	–	Both analogues are mammalian. Significant catalytic core 3D similarity in completely different fold contexts. Divergence followed by convergence is plausible.	[22, 23]
		3.90.190.10	469	2GJT	CDQRT	Thr1143 Cys1136 Arg1142			–		
3‐Hydroxydecanoyl dehydratase	4.2.1.59	3.10.129.110	972	2VZ9	DGHHLY	Any885 Asp1033 Any888 His878	0.62	Yes (27%)	–	One analogue is mammalian, the other is bacterial. Significant catalytic core 3D similarity. Either a case of extreme convergence or distant homology.	[24]
		3.10.129.10	10	1MKA	CDGHV	Any76 Asp84 Any79 His70			–		
Endo‐1,4‐β‐xylanase	3.2.1.8	2.60.120.180	432	2B42	EENYY	Glu172 Glu78 Asn35	1.67	Yes (n/a)	–	Sugar is cleaved with very similar mechanisms using a Glu‐Glu dyad.	[25, 26]
		3.20.20.80	548	1FHD	DEEHN	Glu233 Glu127 Asn169			–		
Peptidylprolyl isomerase	5.2.1.8	2.40.100.10	189	1XO7	FFHLNQR	Any103 Phe62 Phe114	2.56	Yes (n/a)	–	Two Phe residues form a hydrophobic pocket for substrate side chain rotation. Main chain residue is H‐bonded to substrate carbonyl. Both human‐derived, but 362 induces reversible protein folding.	[27, 28]
		3.10.50.40	362	1NSG	DFFIYY	Any56 Phe36 Phe99			–		
Lysozyme	3.2.1.17	1.10.530.10	203	3IJU	DDENNS	Glu35 Asp52 Asp48	2.65	Yes (27%)	–	Hydrolysis mechanism is similar, with slightly different spatial arrangement of residues.	[29, 30]
		3.20.20.80	774	1H09	DDDE	Glu94 Asp92 Asp10			–		
Protein‐methionine‐S‐oxide reductase	1.8.4.11	3.30.1060.10	122	4D7L	CCCDEYY	Asp130 Cys52 Cys206	3.14	Yes (23%)	–	Cys residues have slightly different roles in the two enzymes, but two catalytic steps are similar.	[31, 32]
		2.170.150.20	715	1L1D	CCDHHR	Asp484 Cys440 Cys495			–		
Phosphohistidine‐d‐mannose phosphotransferase	2.7.1.191	1.20.58.80	514	1E2A	HHQ	His78 His82	0.44	Yes (n/a)	–	Two His are at almost identical positions, but reside on different secondary structure elements. Roles in mechanism are similar.	[33, 34]
		2.70.70.10	513	3OUR	HHT	His91 His76			–		
Ubiquitinyl hydrolase 1	3.4.19.12	3.40.532.10	597	4I6N	CDHQ	Any243 His340	1.18	Yes (n/a)	–	Common Cys deprotonation by His with Cys also contributing its backbone in oxyanion hole.	[35, 36]
		2.40.10.10	830	2GZ9	CEGH	Any147 His40			–		
Papain‐like cysteine protease	3.4.22.28	3.90.70.10	805	1Y4H	CHNQ	Any243 His340	1.26	Yes (n/a)	–	Very similar active sites, oxyanion hole is Cys backbone and Ala in one analogue and Cys backbone and Asn in the other. Asn and Glu modify the pKa of the His in both.	[37, 38]
		2.40.10.10	477	6FFS	CEGH	Any147 His40			–		
3‐Dehydroquinate dehydratase	4.2.1.10	3.40.50.9100	55	4KI7	EGHNNPRRY	Glu99 His101	0.83	No (0%)	–	Glu‐His proton relay motif present in both analogues. Glu activates His, but the overall mechanistic context is different.	[39, 40]
		3.20.20.70	54	1QFE	EHK	Glu86 His143			–		

**Table 2:** Analogous enzyme examples with mechanistic‐only convergence.

Enzyme	EC	CATH domain	M‐CSA ID	PDB ID	Residue composition	3D‐aligned residues	RMSD (Å)	Mech. sim. (calc. perc.)	Cofactor	Notes	Refs.
Aminopeptidase	3.4.11.19	3.60.70.12	676	1B65	GNSSY	Ser288 Asn218	1.63	Yes (n/a)	–	Spurious active site similarities, but peptide bond hydrolytic mechanisms share common features.	[41, 42]
		3.40.710.10	782	1HVB	HKNSY	Ser62 Asn161			–		
Chorismate mutase	5.4.99.5	3.30.1330.40	474	1DBF	CERRRRY	Arg116 Arg63 Glu78	4.04	Yes (100%)	–	Active site electrostatic environments and proposed mechanisms are similar, but residues do not superimpose well.	[43, 44]
		1.10.590.10	81	2CSM	EEKNRRT	Arg16 Arg157 Glu198			–		
Ser‐Thr‐Tyr phosphatase	3.1.3.16	3.60.21.10	406	3EGG	DDDHHHHNRR	Asp95 Arg96	1.41	No (0%)	–	Spurious active site similarities but the role of Arg is the same in the two analogues.	[45, 46]
		3.90.190.10	456	5BZX	CDR	Asp92 Arg130			–		
Alcohol dehydrogenase	1.1.1.1	3.40.50.720	255	3W8F	KNSY	–	–	Yes (33%)	NADPH	Highly similar mechanisms, but without detectable structural similarities.	[47, 48]
		3.90.180.10	256	5VKR	CCHHS				NADPH, Zn^2+^		
Histone acetyltransferase	2.3.1.48	3.90.226.10	344	3HE2	AAD	–	–	Yes (40%)	Acetyl‐CoA	Highly similar mechanisms with several overlapping steps. However, no geometrical similarities are detected.	[49]
		3.40.630.30	224	4PSX	EF				Acetyl‐CoA		
Carbonate dehydratase	4.2.1.1 (b)	3.10.200.10	216	5GMN	EHHHHT	–	–	Yes (33%)	Zn^2+^	High mechanistic similarities, no geometrical similarities in active sites.	[16, 50]
		3.40.1050.10	517	5CXK	CCDHR				Zn^2+^		
Carbonate dehydratase	4.2.1.1 (c)	2.160.10.10	516	1QRM	EEHHHNQ	–	–	Yes (44%)	Co^3+^	High mechanistic similarities, no geometrical similarities in active sites.	[15, 50]
		3.40.1050.10	517	5CXK	CCDHR				Zn^2+^		
Cellulase	3.2.1.4	1.50.10.10	559	1KS8	DDEY			Yes (100%)	–	General mechanism of sugar cleavage is the same in all three analogues, involving a water molecule activated by an Asp or Glu residue. Although all three have an acid–acid dyad, active sites do not superpose well probably due to the presence of different bound ligands. Catalytic step order is slightly different between m560 and m561, thus they appear mechanistically different.	[51, 52, 53]
		2.40.40.10	561	3ENG	DD	–	–		–		
		2.60.120.180	560	3VL8	EE			No (0%)	–		
Protein‐glutamate methylesterase	3.1.1.61	3.40.50.180	337	1A2O	DHMST	–	–	Yes (n/a)	–	Methyl‐ester hydrolysis mechanism involves a catalytic triad in both cases, but the nucleophile and stabiliser residues are different.	[54, 55]
		3.30.1330.200	729	2F9Z	CHT				–		
Licheninase	3.2.1.73	3.20.20.80	400	1AQ0	EEEK	–	–	Yes (n/a)	–	Both analogues use an acid dyad to cleave the sugar substrate, but active sites do not superpose well.	[56, 57]
		2.60.120.200	924	1CPM	EE				–		
Penicillin amidase	3.5.1.11	3.60.60.10	241	3HBC	CFNR	–	–	Yes (0%)	–	Similar hydrolysis mechanisms, involving a self‐activated nucleophile and an oxyanion hole in both analogues. Differences lie on the nucleophile (Ser vs. Cys) and the overall catalytic residue arrangement.	[58, 59]
		3.60.20.10	841	1GK9	ANS				–		

**Table 3:** Analogous enzyme examples with reaction‐only convergence.

Enzyme	EC	CATH domain	M‐CSA ID	PDB ID	Residue composition	3D‐aligned residues	RMSD (Å)	Mech. sim. (calc. perc.)	Cofactor	Notes	Refs.
Acid phosphatase	3.1.3.2	3.60.21.10	43	1KBP	DDHHHHHHNY	His296 Asp164 His325	1.40	No (0%)	Zn^2+^, Fe^3+^	Every pairing of analogues has active site similarities and in every case it involves ligand/cofactor binding residues (different residues match in every pair). Purple acid analogue uses metal cofactors, and the other two were crystallised with metal ions to substitute the phosphate substrate. Mechanisms share some similarities within individual pairs but are generally different.	[60, 61, 62]
		3.40.50.1240	454	4JOC	DHHRRR	His59 Asp335 His334			–		
						Arg168 His334 His59	1.98	Yes (n/a)			
		1.20.144.10	558	1EOI	DHHR	Arg183 His189 His150			–		
		3.60.21.10	43	1KBP	DDHHHHHHNY	His189 Asp193 His150 His325 Asp135 His295	1.48	No (0%)	Zn^2+^, Fe^3+^		
Phospholipase A2	3.1.1.4	3.40.1090.10	529	5IZR	DGGRS	Any330 Asp647	2.56	Yes (23%)	–	Spurious active site similarities. Mechanisms are different.	[63, 64]
		1.20.90.10	528	1FAZ	DDDHL	Any44 Asp85			Ca^2+^		
Catalase	1.11.1.6	1.20.1260.10	572	1JKU	EEEEHH	His69 His181	4.31	No (n/a)	Mn^3+^	Spurious active site similarities. Mechanisms are different.	[65, 66]
		2.40.180.10	573	3VU3	HHN	His392 His128			Heme		
Chloride peroxidase	1.11.1.10	1.10.606.10	14	1IDQ	GHHKRRS	Ser402 His404 Any403	2.98	No (0%)	VO_4_	Any calculated active site similarities are spurious amongst the three analogues. Mechanisms are completely different, and the only commonality is Cl^−^ selectivity, which is one of three substrates (Cl^−^, H_2_O_2_ and a variable organic compound that is chlorinated).	[67, 68, 69]
		3.40.50.1820	248	1BRT	DFHST	Ser98 His257 Any32			–		
						Asp228 His257	2.86	No (0%)			
		1.10.489.10	250	2CJ0	CDEH	Asp106 His105			Heme		
Β‐lactamase	3.5.2.6	3.60.15.10	258	6U13	DHHHHHY	–	–	No (0%)	Zn^2+^	Catalysis is metal‐dependent in one analogue, in contrast to the other.	[70, 71]
		3.40.710.10	210	1M6K	ASSSWX				–		
Dihydrofolate reductase	1.5.1.3	2.30.30.60	752	1VIF	1KQY	–	–	Yes (n/a)	NADPH	No similarities in mechanism detected, however, both analogues use NADPH for hydride transfer to dihydrofolate.	[72, 73]
		3.40.430.10	490	1DHF	EL				NADPH		
Pectate lyase	4.2.2.2	2.160.20.10	896	1O8H	DDDER	–	–	No (n/a)	Ca^2+^	The only mechanistic similarity lies in the presence of an Arg proton shuttle, however, the overall mechanism differs. The bacterial analogue also requires a calcium activator of Arg.	[74, 75]
		1.50.10.20	509	1GXN	NR				–		
Cellulose 1,4‐β‐cellobiosidase	3.2.1.91	3.20.20.40	440	1QK0	DDDRSY	–	–	No (n/a)	–	Both analogues cleave cellulose to celloctriose, with different mechanisms (invertin vs. retaining).	[76, 77]
		2.70.100.10	444	6GRN	DEEH				–		
ADP‐ribosyltransferase	2.4.2.36	3.90.175.10	773	1TOX	E	–	–	No (n/a)	–	Toxin mechanisms are not well studied. The analogues both use NAD and dipthamide as substrates but are likely unrelated.	[78, 79]
		3.90.210.10	919	1XTC	EERS				–		

### Paradigms of convergent evolution

Thirty‐four cases of isozymes in M‐CSA reveal three paradigms of functional convergence, defined below:

1. *Structural and mechanistic* (Table 1). Two or more catalytic residues align in 3D, and there are similarities in the mechanism. This is a very common paradigm, with 14 examples. In some cases, a subset of catalytic residues may align in 3D, but the mechanisms are generally different. This situation is not common here (2 examples), and such similarities are usually located on a ubiquitous motif (e.g. an activator stabilised by an acid or an ion binding motif).
2. *Mechanistic‐only* (Table 2). Similarities in mechanism are observed, with no aligned catalytic residues. This is also a very common paradigm (11 examples). In such cases, a general similarity in the active site might exist, but this is not detectable in superposition.
3. *Reaction‐only* (Table 3). Only the overall reaction is similar, with no similarities in mechanism or active site geometry. A moderately common paradigm for which nine examples are found.

Within all paradigms, analogues might: (a) use similar cofactors, (b) use different cofactors or (c) one uses a cofactor, while the other does not. There are also ambiguous cases in this dataset. For instance, chorismate mutases (EC 5.4.99.5) can be assigned to either paradigm 1 or 2, depending on how structural and functional similarities are interpreted.

### Active site 3D similarities accompany mechanistic similarities (Table 1)

Several examples in our data correspond to mechanistic analogues, with similarities in mechanism being reflected in 3D (structural and mechanistic convergence).

**Fig. 3:**
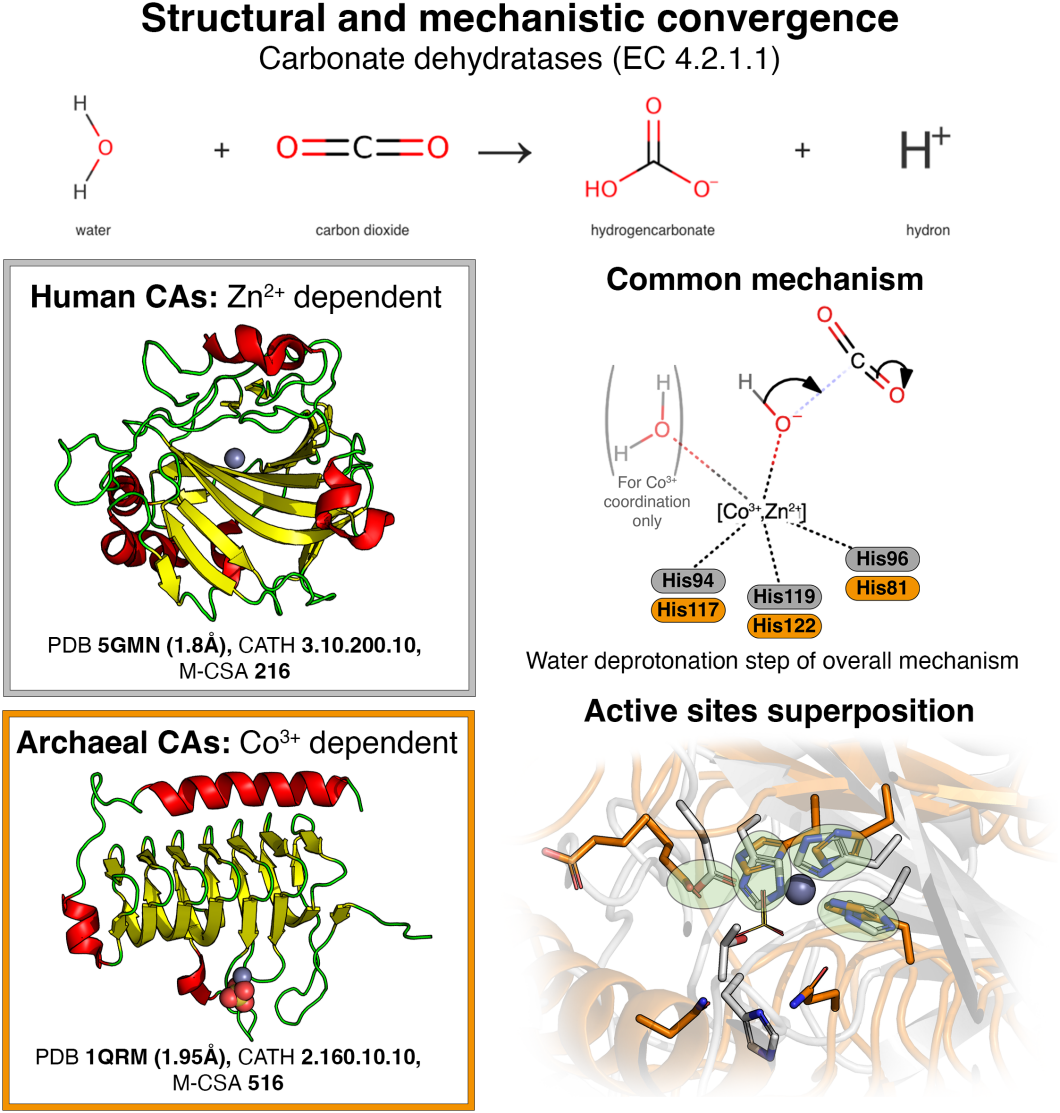
Structural and mechanistic convergence between human (grey) and archaeal (orange) carbonate dehydratases. The two similar mechanisms are shown as a consensus mechanism in the middle right part of the figure. Green circles in the active sites' superposition indicate pairs of 3D‐aligned catalytic residues (bottom right part of the figure).

To illustrate this paradigm, we selected the human and yeast carbonate dehydratase analogues. These classes of carbonate dehydratases (alpha and gamma) have converged to catalyse the reversible conversion of carbon dioxide and water into hydrogencarbonate (Fig. 3). They use a similar mechanism where the most important catalytic step is a nucleophilic attack on the carbon dioxide by a hydroxide ion, which is being stabilised by a positively charged metal ion (mechanism step picture in Fig. 3). Interestingly, although the enzymes use a different metal ion, Co^3+^ in the case of the gamma class and Zn^2+^ for the alpha class, both active sites provide three histidine residues to hold the metal ion in place. While the coordination sphere of Zn^2+^ is satisfied by the three histidines and the hydroxide, Co^3+^ binds an additional water molecule and as the reaction proceeds, the hydrogencarbonate product. The active sites also differ in the residue that deprotonates the water molecule to generate the hydroxide (a His for alpha vs. a Glu for gamma) and the residues that orient the carbon dioxide (Glu and Thr for alpha class vs. Asn for gamma class).

**Fig. 4:**
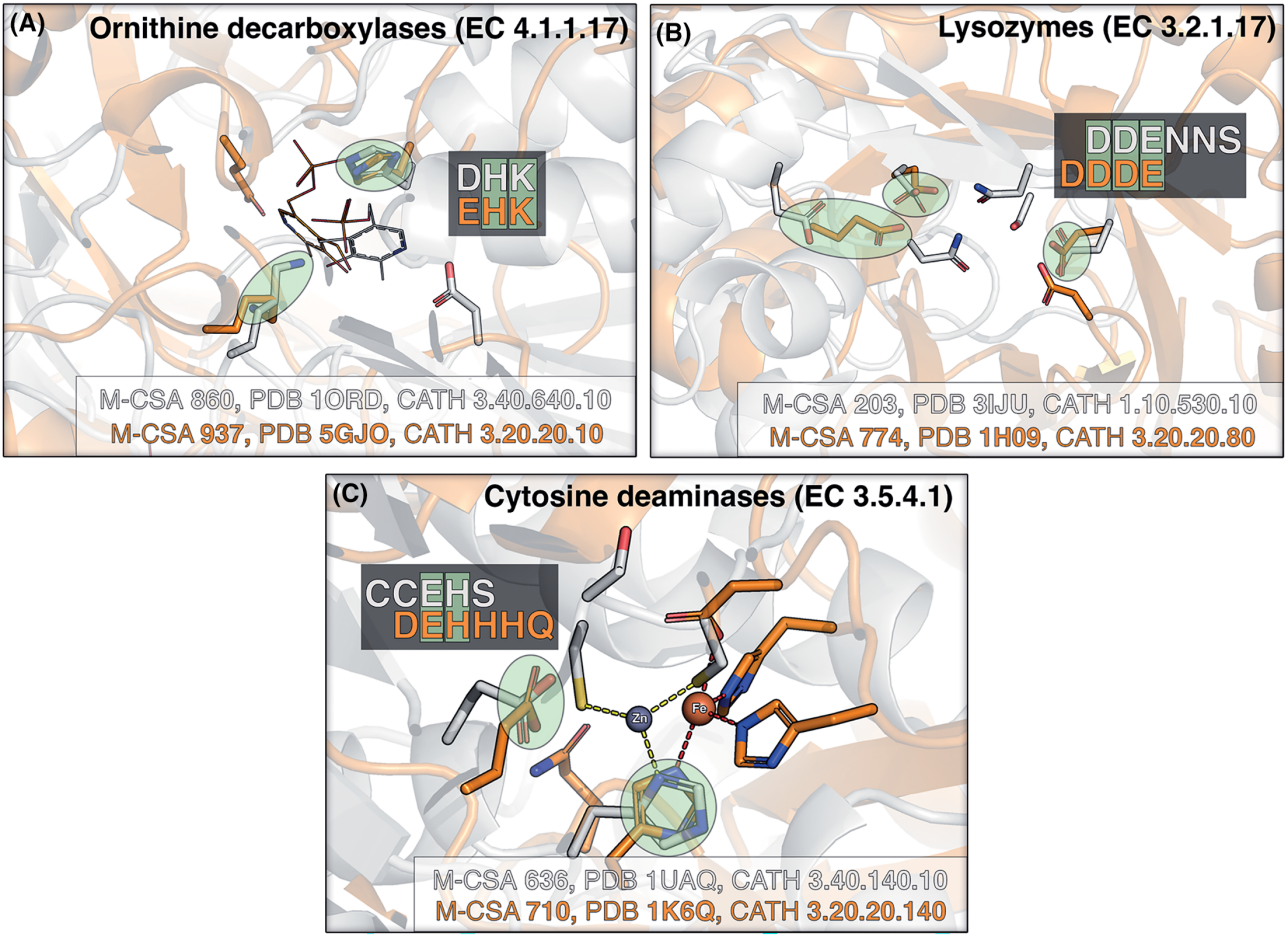
Active site similarities in three pairs of analogues with structural and mechanistic convergence. (A) Bacterial (grey) and mammalian (orange) ornithine decarboxylases. (B) Chicken (grey) and viral (orange) lysozymes. (C) Yeast (grey) and bacterial (orange) cytosine deaminases. Green circles indicate 3D‐aligned catalytic residue pairs. Residue alignment is also shown on the active site residue composition (pseudo‐sequence as in Table 1) with the aligned residues highlighted in green.

Another illustrative example is the pair of mammalian/bacterial ornithine decarboxylases (EC 4.1.1.7) [80] that both use an (Asp/Glu)‐His‐Lys residue triad to cleave a carboxylate from the ornithinium substrate (Fig. 4A). A PLP cofactor is involved in both mechanisms that is initially covalently bonded to the catalytic Lys. Global sequence alignment showed low similarity although the local alignment has relatively high coverage. The enzymes have no overall structural similarity, belonging only to the same primary CATH class (1st level). In their active sites, the two analogues have mirrored but a similar arrangement of catalytic residues, with Lys and His endpoint atoms superposing at RMSD 1.75 Å. By these observations, homology is unlikely; instead, convergence in function, local structure and mechanism is inferred.

Structural constraints may be relaxed in some cases, with slightly different functional atom 3D configurations facilitating the same mechanistic sequence. Mammalian and viral lysozyme analogues (EC 3.2.1.17) are a good example (Fig. 4B), in which the carbonyl groups of three acidic residues (one Glu and two Asp) loosely superpose at RMSD 2.65 Å. Glycosyl hydrolases GH22 (viral) and GH25 (mammalian) break down β‐MurNAc‐(1 → 4)‐β‐d‐GlcNAc to *N*‐acetyl‐β‐d‐muramic acid and *N*‐acetyl‐β‐d‐glucosamine via hydrolysis, with two proposed mechanisms in each analogue. They have virtually no structural similarity, and GH25 uses a choline ion to facilitate cell wall binding, which is not directly involved in catalysis but does increase catalytic activity, while GH22 does not use any cofactors. Global and local sequence alignments showed low similarity, and except for Glu35 in GH22 lining up with a noncatalytic Glu in GH25 in the global alignment, no active site residues line up with an identical (or near‐identical) residue in any alignment. The only differences between them are in which atoms serve as donors/acceptors, and in one case the active site regeneration chemistry is different. Every proposed mechanism directly involves a Glu with an intramolecular O atom (the latter is present in three out of total of four proposals) to break the glycosidic bond. Both have the presence of two acidic residues hugging the substrate, similar to cellulases. The analogues show 27% mechanistic similarity, and given the low sequence and structural similarity, it is likely that they evolved this function separately.

Evolution can also shape active sites in a modular manner, with resulting enzymes having similarities in the mechanism, yet still differing slightly. A good example is cytosine deaminases (EC 3.5.4.1) where both analogues use a catalytic metal, coordinated by different residue sets (Fig. 4C): 3 His and an Asp binding a Fe ion in the yeast enzyme, while 1 His and 2 Cys bind a Zn ion in its bacterial analogue. In both cases, the metal binding His and the proton donor/acceptor Glu have a similar 3D arrangement, with the latter protonating the cytosine N3 by obtaining a proton from a water molecule. Within this paradigm, we distinguish analogues that have obscure similarities, located in motifs such as ion binding sites or activator‐stabiliser dyads (e.g. His‐Glu or His‐Asp). This is seen in two examples, both containing a His‐acid dyad: 3‐dehydroquinate dehydratases (EC 4.2.1.10) and endonucleases (EC 3.1.21.1). In all those, the acid (Asp/Glu) is hydrogen‐bonded to the histidine, to activate and/or sterically orientate it, in different mechanistic contexts. The same dyad also exists in catalytic triads of Ser/Cys proteases with the acid playing the same role.

### Mechanistic similarities with different catalytic residues (Table 2)

We observed cases where mechanistic similarities might exist, without detectable active site similarities (mechanistic‐only convergence). In such cases, any structurally aligned residues found by our alignment algorithm are most likely spurious or superpose loosely. This is demonstrated in fungal and bacterial chorismate mutases (EC 5.4.99.5), between which three catalytic residues superpose at a very high RMSD (4.04 Å—considered dissimilar), but the overall electrostatic environment that facilitates isomerisation of the chorismate substrate is similar by visual inspection.

**Fig. 5:**
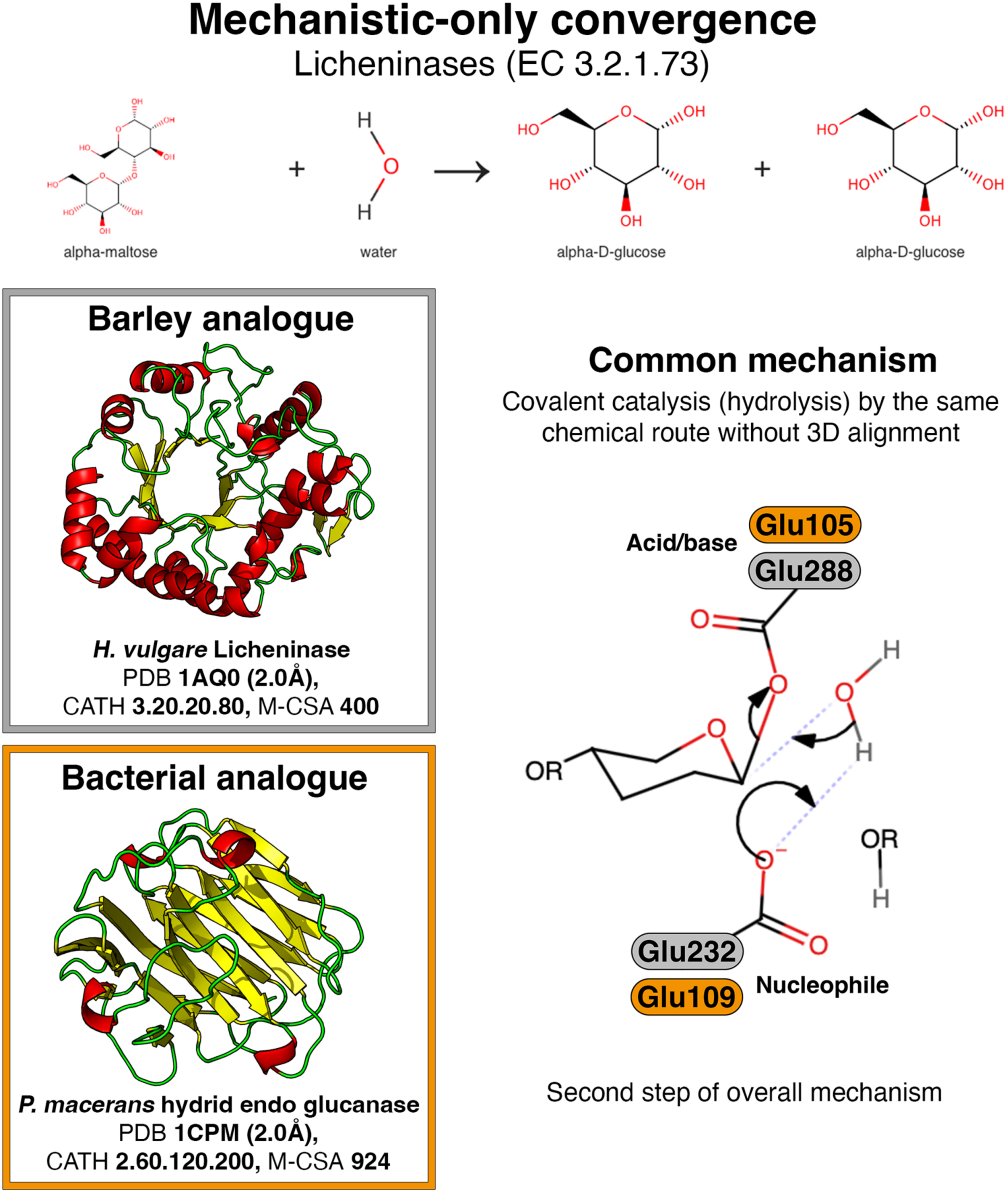
Mechanistic‐only convergence between plant (grey) and bacterial (orange) licheninases. Although there is no structural alignment in the global or local level, the mode of action of both active sites is similar, mediated by two acidic residues between which the substrate enters to be hydrolysed (mechanism diagram in the bottom right part of the figure).

A representative example of the ‘mechanistic‐only’ paradigm is licheninases (EC 3.2.1.73) produced by both plant and bacteria for cell wall degradation. These are unrelated in primary sequence (20% sequence identity) and tertiary structure (Fig. 5). Despite the differences in protein fold and local geometry they retain a similar mechanism for cleaving cereal β‐d‐glucans and lichenin with the formation of an intermediate covalent bond between the substrate and a glutamic acid in the enzyme during catalysis [56].

Aminopeptidase analogues from *Ochrobacterium anthropi* (EC 3.4.11.19) also conform to this paradigm. They are also a great example of a duplicate function within the same organism/cell/compartment, presenting the evolutionary question of why two analogous enzymes would co‐exist. The two analogues are known as DmpA (M‐CSA entry 676) and DmpB (M‐CSA entry 782) and show no evidence of a common ancestor. However, their mechanisms are similar without geometrical convergence in the active site. DmpA is poorly characterised, mostly because it is expressed in low quantities [81]. DmpB is more abundant and better characterised and is also an ancestor of penicillin resistance‐conferring enzymes (β‐lactamase activity) [42]. It is plausible that the two analogues co‐exist in the same organism, because they are regulated differently. DmpA is also functionally promiscuous, with d‐esterasic and d‐amidasic activities as well as autoproteolysis [43], suggesting that aminopeptidase activity is not the primary function compared to DmpB, whose function is more specific.

### Convergent evolution with different catalytic residues and mechanism (Table 3)

Enzymes can catalyse the same overall reaction, but with different mechanisms and active site structures (reaction‐only convergence). This paradigm is moderately abundant in M‐CSA (9 examples) and refers to enzymes that have the same EC number (i.e. they catalyse the same reaction) but have no detectable similarities in their overall or active site 3D structure.

**Fig. 6:**
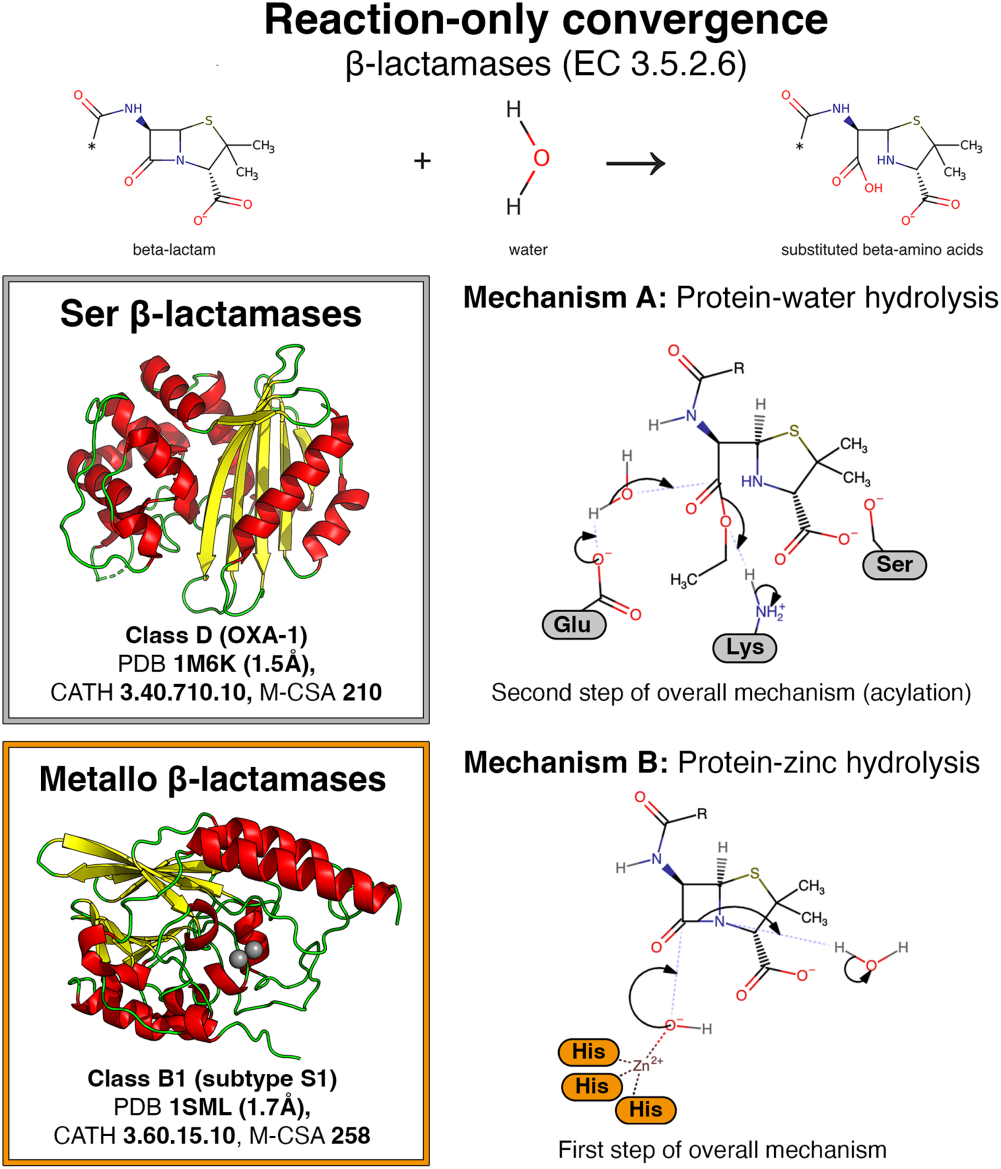
Reaction‐only convergence in β‐lactamases. The exact acid and bases, and the sequence of catalytic events vary in different Ser β‐lactamases. Metallo β‐lactamases bind two metal ions in the active site, but only the one participating in the described steps is shown in the mechanism diagram.

In the case of fungi, for example, the two cellobiosidases (EC 3.2.1.91) act on the cellotetraose substrate using a different ensemble of residues and mechanisms (inverting vs. retaining [82]) for bond cleavage, giving isomeric products of opposite stereochemistry [83]. Similarly, structurally different β‐lactamases (EC 3.5.2.6) in bacteria perform the same catalytic function using either a protein‐water or a distinct protein‐metal mediated process (Fig. 6). Reaction‐only convergence can also be observed across taxa, for example, the peroxidases (EC 1.11.1.10) from fungus and bacteria (Table 3). In this case, the oxidation of the substrate is achieved by utilising significantly different mechanisms through the selective exploitation of nonproteinous components like chlorine, haeme or vanadium.

### Convergence and distant divergence are often indistinguishable

Convergence is not easily distinguished from distant divergence, especially in the case of generic metabolic enzymatic activities. Such analogues have little sequence and overall structural similarity, belonging to different CATH superfamilies (H level) or even having different topologies (T level) and secondary structure architecture (A level). However, their active/binding sites and overall conformation of the catalytic core occasionally retain structural similarity.

This is exemplified in two mammalian tyrosine phosphatases (EC 3.1.3.48) that have a remarkably similar catalytic core but completely different overall folds (CATH 3.40.50.2300 for the bovine low molecular weight phosphatase, vs. CATH 3.90.190.10 for the human tyrosine phosphatase). For these two analogues, Gherardini *et al*. suggest convergence [3], however, the striking similarities in the vicinity of the active site could infer two additional plausible scenarios: (a) divergence to accommodate different metabolic needs and subcellular location (endoplasmic reticulum vs. cytoplasm), with conservation of the catalytic core, or (b) divergence towards a different function, followed by re‐convergence. Evolutionary linkage is further supported by the same sequence order of the three aligned catalytic residues (Ser20‐Cys13‐Arg19 vs. Thr1143‐Cys1136‐Arg1142). Another example of evolutionary ambiguity is the pair of two bacterial/mammalian 3‐hydroxydecanoyl dehydratases (EC 4.2.1.59) that share high similarity in the extended active site, with four catalytic residues superposing at very low RMSD (0.62 Å). This is accompanied by high mechanistic similarity. Our observations lead us to believe these analogues are either an extreme case of convergent evolution or a case of horizontal gene transfer from symbiotic bacteria to the host.

### Convergent evolution and EC classification

We identified enzymes of similar EC classification that have substantial functional differences.

Although we are looking at identity in all four EC levels, which should imply identity in substrate selectivity, this is fuzzy in some enzymes, such us polymer‐acting ones, or ones using multiple substrates, like chloride peroxidases (EC 1.11.1.10). Three analogues in our set share this EC and are totally unrelated. Structures, mechanisms and metabolic contexts are completely different, and their only commonality is selectivity for Cl^−^ ions and H_2_O_2_. The third cognate substrate is a variable organic compound that is chlorinated. Reactions like this are generic and involve several components, one of which is simplistic (like an ion). Also, Markush structures are often used to represent ensembles of substrates that share a common chemical group. In such cases, EC often classifies the reaction based on the simplest component. This means that functional differences might be concealed. Similar ambiguities are observed in promiscuous enzymes, such as proteases or DNAses, leading to the same problem. Additional complexity in EC classification can derive from the type of cognate ligand considered for defining the fourth number. In most hydrolases, for example, phosphatases from the 3.1.3.x sub‐subclass, the sub‐sub‐subclass is defined by the substrate, whereas in triterpene synthases (5.4.99.x), it is defined by the product.

## Discussion

We have collected a sample of enzyme analogues with sufficient structural and functional evidence, to reveal some ‘rules’ or paradigms of convergent evolution. These data, albeit limited, have allowed us to identify cases of chemical convergence in enzymes (catalysis of the same overall reaction), accompanied by structural convergence in the active site and mechanistic convergence. We showed that these three levels of similarity might not necessarily co‐exist, and based on this, we defined three paradigms of convergence: structural and mechanistic (13 cases), mechanistic‐only (11 cases) and reaction‐only (9 cases).

Classification of analogous enzyme groups into paradigms was performed in a semi‐manual way, by calculating quantitative measures of similarity (catalytic residue superposition and mechanistic similarity), and by referring to available literature. In this process, we realised that the quantitative measures alone are not sufficient for assignment into paradigms. Therefore, each case was examined individually and if necessary, the quantitative results were overridden. For example, there were several cases where geometrical similarities were found to be spurious, and the active sites had to be examined manually to ensure biological relevance. Interestingly in the context of template matching: we have found that smaller templates, consisting of a few atoms, often yield spurious matches, even if the search is restricted to active sites. Therefore, one needs to be aware of false‐positive results in template‐based searches, especially when these are performed at a high‐throughput level.

Our methodology also included an examination of cofactor selectivity. If we investigate the paradigms further by considering cofactor presence, there were four unobserved scenarios in this survey. One case refers to enzymes with similar active site geometries, dissimilar mechanisms and the same bound cofactor. This could be because having these traits in common is usually indicative of a homologous relationship. On the other hand, we also did not observe any enzymes that had similar active site geometry and mechanism, but significantly different cofactors (e.g. a metal vs. organic cofactor); this is also expected as the presence of cofactors would affect the geometry and mechanism of the active site. We also do not see a pair of enzymes with similar active site geometries and mechanisms, where one enzyme uses a cofactor, and one does not. This is also expected for the same reason as previously; however, we observe this when mechanisms differ (e.g. in acid phosphatases). Although there are examples where a reaction can be performed under both a cofactor‐dependent and ‐independent mechanism, these are mostly classified into the ‘reaction‐only’ paradigm. This is probably because it is quite unlikely that enzymes would have the same geometry and cofactors, without having any mechanistic commonality. Overall, these results could imply some level of rigidity that comes with active site geometries being similar. Lastly, we do not see any cases where two enzymes with similar mechanisms have significantly different cofactors. This could be attributed to the limited variety of cofactors in nature, where each cofactor has evolved to serve a specific role in catalysis [84]; thus, it is unlikely for two enzymes to evolve to bind different cofactors and still have a similar mechanism.

To address the question of why solutions to chemical catalysis have evolved several times, we have found several examples of comparable chemical reactions performed by analogous enzymes. These data indicate that the factors influencing convergent chemistry between nonhomologues enzymes are as follows: (a) a common need for a metabolic reaction, but independent evolution has led to the emergence of different molecules. This is mostly observable in analogues from different kingdoms of life; (b) a different metabolic context demanding different enzymes; (c) different analogues might be regulated differently; (d) environmental pressures to solve biological challenges with the limited raw material available (i.e. amino acids, water, metals, etc.) leading to similar solutions, with the more efficient being retained. We should add here that M‐CSA enzyme coverage, and consequently this survey, is limited to soluble proteins, as there is more abundant structural evidence for them compared to membrane proteins. However, we do acknowledge cases of functional similarities between soluble and membrane‐bound enzyme analogues. Two examples are (a) the DkgA/DkgB pair (mammalian membrane and bacterial soluble respectively), two mechanistically similar diacylglycerol kinases that share active site similarities [85]; and (b) soluble and membrane urate oxidases (Uox), that catalyse the same reaction albeit with different mechanisms [86]. Furthermore, our study does not consider kinetic parameters that might differ radically between analogues, providing catalysis in different metabolic contexts that require different reaction rates. This phenomenon also applies to promiscuous enzymes, where secondary functionalities might be slower, a topic discussed in our recent review [1].

Analysing how proteins with completely different folds maintain identical catalytic activity, we found many cases where a reaction might be mechanistically and/or structurally constrained. The more constrained the process, the higher the similarities between analogues (e.g. phosphatases). Other processes are less constrained, and convergence is only observable at the end of the reaction (for instance, metal‐dependent vs. metal‐independent β‐lactamases, see Fig. 6). It is also worth noting that enzymes that use the same metal ion as a cofactor, often share common geometry in the ion binding site. This has been extensively discussed in our previous work on active site modularity [6]. Our approach to examining convergence from various points of view was hybrid, including both manual inspection and automated/quantitative methods. The latter can be scaled in a reproducible pipeline, allowing a more systematic and comprehensive analysis of convergence in the future, not necessarily restricted within the limits of M‐CSA. In the interest of clarity and conceptualisation, we have generalised the observations from the data, where each pair of enzymes evaluated provides unique insights into how proteins can maintain the same catalytic potential on a completely different overall shape.

## Materials and methods

### Data preparation

Groups of enzymes catalysing the same reaction and having different folds were collected from M‐CSA [87] using the public API, by querying for entries with identical EC numbers and different CATH [88] numbers in the catalytic domain of their reference structure. Entries were grouped by EC number, and those groups having redundant CATH numbers, multiple CATH numbers (e.g. active site formed in the interface of two domains), or incomplete information (not all 4 EC levels determined, no CATH mapping, etc.) were removed. In cases of EC groups having more than two enzymes after filtering, relationships were analysed in pairs, so that groups with three enzymes would result in three distinct analyses. This first filtering resulted in 46 pairs of enzymes that perform the same function and have different folds, across 34 unique EC numbers. Some extra filtering was done to ensure that this survey focuses solely on pairs that have similarities due to functional convergence. From the original set of 46 pairs, four were excluded as sequence or structural similarity implied potential homology. Pairs from the restriction enzyme group (EC 3.1.21.4, accounting for six pairs) were also excluded for the same reason [67, 68, 69]. Five pairs were taken out due to limited available information. In several cases, two enzymes may not bind identical substrates or products, but those are still in scope since some of them are substrate promiscuous. Most of these were due to unspecified ‐R groups, different substrate specificities, or both enzymes manipulating the same functional groups on different molecules. The final, reduced set included 31 pairs across 23 EC numbers. Twenty‐one out of the 31 pairs were found to have mechanism similarity, however, none of the pairs showed complete identity.

### Literature analysis

Each pair was analysed using the information found on M‐CSA (enzyme mechanism and catalytic residue reaction roles), PDBe [89] (representative crystal structure selection, assembly composition), PDBsum [90] (bound ligand information) and the enzymes associated literature from these databases.

### Sequence and structure comparison

Local and global sequence alignments were performed using the Needleman‐Wunsch and Smith‐Waterman algorithms, respectively, via the EMBOSS service [90, 91]. Alignments were visualised in jalview [92].

### Active site structure comparison

Functional atom templates [9] (three atoms per residue) were generated for each active site, taking all possible combinations of two, three and four residues [93]. Template definitions are mechanism‐informed (e.g. three backbone atoms are selected for residues known to contribute their backbone to the mechanism) and allow for alternative matching of chemically similar atoms and residues, as described in Ref. [6]. Templates of the same size were structurally compared using the template matching programme Jess [94], in an ‘all against all’ fashion. The match with the lowest RMSD and highest number of residues was selected as a reference atom–atom mapping frame for dynamic active site fitting.

### Dynamic active site fitting

Using the active site atom–atom mapping method described above, enzyme pairs were fitted on their functional atoms, using a Gaussian‐weighted version of the Kabsch algorithm [95, 96], as described in Ref. [93]. The algorithm outputs the two enzymes with their coordinates transformed accordingly, along with a weighted RMSD (wRMSD), an unweighted RMSD and a coverage value. The latter corresponds to the proportion of mapped catalytic residues over the number of residues of the largest of the two active sites.

### Catalytic mechanism comparison

Mechanism comparison was conducted, which resulted in pairs being categorised as having ‘similar’ or ‘not similar’ mechanisms which were determined by seeing if the enzymes had any similarities in their mechanism. Similarities included near identical steps (ex: nucleophilic attack on the same atom, not necessarily the same nucleophile) or identical order of some or all events. This was initially done by manual inspection and comparison of the mechanisms found on M‐CSA, and in cases where both enzymes in a pair had detailed mechanism description, a mechanism similarity score was calculated as follows: the data on the curly arrow diagrams of each catalytic step are first abstracted into a set of curly arrows and their chemical environments. This representation of each curly arrow is called an arrow environment and it includes the atoms interacting directly with the curly arrow and two shells of atoms and bonds around those. The similarity score for each mechanism pair is then calculated as the Jaccard index of the two sets of arrow environments (unpublished data).

### Cofactor binding comparison

Cofactor preferences were compared for each enzyme pair, focusing only on cofactors directly involved in catalysis. The result of this analysis was placement into two broad categories that addressed identity, with pairs having the same cofactors being marked ‘yes’ and ones with different cofactors being marked ‘no’. Within the ‘yes’ and ‘no’ categories there were subcategories. In the ‘yes’ category pairs were marked as both having present cofactors that were identical, or both having no cofactor present. In the ‘no’ category pairs were marked as both having cofactors that are different, or one having a cofactor present and one not having a cofactor present. We considered NAD and CoA cofactors if they were in the reaction regardless of their original chemical composition was regenerated at the end of the catalytic cycle.

## Conflict of interest

The authors declare no conflict of interest.

## Author contributions

IGR contributed to conceptualisation, methodology, software, validation, writing—original draft preparation. JCK contributed to methodology, investigation, visualisation. GO contributed to software, methodology. AJMR contributed to data curation, validation. NB contributed to conceptualisation, validation, writing—review & editing. JMT contributed to conceptualisation, supervision, resources, funding acquisition, project administration, writing—review & editing.

### Peer review

The peer review history for this article is available at https://www.webofscience.com/api/gateway/wos/peer‐review/10.1111/febs.17332.

## Data Availability

The data that support the findings of this study were derived from the following resources available in the public domain: M‐CSA for enzyme mechanism and active site residue annotations (https://www.ebi.ac.uk/thornton‐srv/m‐csa/), UniProt for protein sequences (https://www.uniprot.org) and PDBe for protein structures (https://www.ebi.ac.uk/pdbe/). Jess [94] is available at https://github.com/iriziotis/jess and its Python implementation pyJess is available at https://github.com/althonos/pyjess. The mechanism similarity software is soon to be documented and released.
